# Magnetorheological Fluids Actuated Haptic-Based Teleoperated Catheter Operating System

**DOI:** 10.3390/mi9090465

**Published:** 2018-09-13

**Authors:** Xuanchun Yin, Shuxiang Guo, Yu Song

**Affiliations:** 1 College of Engineering, South China Agricultural University, 483, Wushan Road, Tianhe District, Guangzhou 510642, China; xc_yin@hotmail.com; 2 Faculty of Engineering, Kagawa University, 2217-20 Hayashi-cho, Takamatsu 760-8521, Japan; guo@eng.kagawa-u.ac.jp; 3 Graduate School of Engineering, Kagawa University, 2217-20 Hayashi-cho, Takamatsu 760-8521, Japan

**Keywords:** magnetorheological fluids, haptic interface, catheter minimally invasive surgery

## Abstract

During conventional catheter endovascular procedures, surgeons needs to adjust the catheter intervention moving direction and velocity according to the direct sensation. Moreover, in the conventional method, both the surgeon and the patient are inevitable exposed to a large amount of, and for a long period of time, X-ray radiation during the surgical procedure. The purpose of this paper is to ensure surgical safety and to protect the surgeon from X-ray radiation during the surgical procedure by adopting a novel haptic-based robot-assisted master-slave system mode. In this paper, a kind of magnetorheological fluids (MR fluids)-based haptic interface has been developed to generate a kind of controllable haptic sensation providing to the catheter operator, and the catheter intervention kinematics parameters measured the motion capture part to control the salve robotic catheter operating system following the master side kinematics. The slave catheter operating the mechanical system has also been designed and manufactured to manipulate the clinical catheter by mimicking the surgeon operating the catheter intervention surgical procedure, which has a 2-DOF (advance, retreat, and rotate) catheter motion characteristic; in addition, the interaction force between the catheter and inner wall of vasculature can be measured by its force sensing unit and the feedback to the master system. The catheter intervention synchronous evaluation experiments between the master and slave system are tested. Also, the advantages of integrating the controllable haptic sensation to the master-slave system experimental evaluations have been done in vitro. The experimental results demonstrated that the proposed haptic-based robot-assisted master-slave system mode can reduce the surgical time and protect the surgeon from X-ray radiation.

## 1. Introduction

Cardiovascular diseases are the major cause of mortality in developed countries, causing roughly 34% of deaths each year [1]. The treatment of these diseases has gone from open surgery techniques to minimally invasive intervention advent with technological development. Minimally invasive endovascular surgery is accomplished through the use of catheters and guidewire that are inserted percutaneously via the artery into target lesions. In conventional endovascular surgeries, however, in order to insert catheters and guidewire through the vasculature system of the body, the physician has to rely on visuals obtained from vascular electrocorticography (using pre-implant magnetic resonance imaging (MRI) and post-implant computerized tomography (CT) co-registration) and the sensed forces at the fingertips [2], as well as being exposed to large doses of longtime X-ray radiation during the surgery. Successful surgery mainly depends on catheter navigation skills and feedback. The navigation skills include the direction of and the velocity of insertion, retraction, and rotation of the proximal end of the catheters and guidewire [3,4]. Especially at the vessel branches where the tip of the catheter moves from the main branch (the bigger diameter) into the minor branch (the smaller diameter) skill is needed.

Compared with conventional endovascular surgeries, the advantage of minimal catheter interventional surgery has been accepted by the public, such as short recovery time, a small incision to the health issue, good surgical outcomes and little post-operative pain have facilitated in the adoption of endovascular surgical techniques [5]. However, for physicians, long fluoroscopy times and exposure to X-ray radiation lead to problems like cancer and cataracts. Even though lead aprons are worn, the face and hands are still exposed to X-ray radiation, and, the heavy protection suits coupled with long hours of standing during the surgical procedure can lead to neck and back pain, as well as injury [6]. Moreover, with manual minimally invasive surgery (MIS), many surgeons may suffer from experience limitations such as hand tremors, fulcrum effect, and physical fatigue during the surgical procedure [7]. In this paper, in order to solve these disadvantages and limitations that exist in MIS, a kind of haptic-based robot-assisted master-slave surgical system mode was presented.

However, most mature surgical scenarios are equipped with robot-assisted master-slave system. These include, da Vinci (Intuitive Surgical, Sunnyvale, CA, USA [8]), Raven and Raven II (Washington University, Washington, DC, USA [9]), insertable robotic effector platform IREP (Columbia University, New York, NY, USA [10]), and some others. In their robot-assisted surgery scenario, the surgeon does not control the slave system directly but remotely manipulates the remote robotic commands input interface, which is called the master system. The master system has the function of measuring the surgeon’s operating kinematics during the whole surgical procedure which are used to control the slave robot manipulator. In essence, it is a kind of input device directly operated by the surgeon. So, the design of the master system should take care of following several requirements such as adequate workspace, minimal back-drive friction, low inertia, reduced backlash, and ergonomic features among others [11].

However, the absence of haptic feedback has been considered a major drawback in these robot-assisted master-slave surgical systems due to physical separation between the surgeon and the patient [12]. Several studies have illustrated the important aspects of the haptics-based teleoperated robot-assisted surgical system, especially in terms of task accuracy and force. In these researches, stability and transparency were viewed as the most important characteristics; the role of damping in stability criterion analysis for haptic rendering is presented in [13]; transparency of the master-slave system research was studied in [14]; inherent stability and transparency issues in haptic interaction can be solved by establishing human arm impedance modelling, control strategy and the related literature review summarized in [15]. Except for stability and transparency, telepresence is viewed as one of the most sophisticated technologies that allows the separation of individuals from the teleoperated working environment and has been realized by haptic augmentation strategies [16]. Therefore, providing haptic cues to the operator in the stable master-slave system promises to ease task execution and increase control accuracy.

As described above, it is necessary to provide haptic sensation in robot-assisted master-slave surgical system mode as well as keeping system stability. However, two main challenges exist in developing compelling haptic interfaces. One challenge is how to generate a kind of controllable haptic sensation. The designed haptic interface should exhibit low intrinsic friction and inertia to minimize dynamic distortion of the user’s perception and to be controlled. Meanwhile, operation dexterity and habituation also need to be considered in haptic interface design especially in a surgical application scenario. The critical factor in controllable characteristics of a haptic interface design is the haptic actuator chosen and the structural design of the mechanism. Traditionally, most haptic devices mainly rely on electric motors, pneumatic actuators, or electric motors and pneumatic integration.

It is worth pointing out that magnetorheological fluids (MR fluids), a kind of smart material, has been chosen as an actuator in haptic interface design due to viscosity controllable characteristics such as: Experimental performance of small-scale MR fluid-based clutches in haptic interface was evaluated in [17]; the multi-finger interface is equipped with three MR fluids actuator mechanism developed in [18]; MR fluids based on multi-degree motion haptic mechanisms design in [19], these researches focus on the haptic interface design by utilizing MR fluids. Besides these, some control method has been presented in experimental evaluation of a miniature MR device for a wide range of human perceivable haptic sensations [20], and virtual surface characteristics of a tactile display by utilizing MR fluids [21]. Therefore, in this paper, MR fluid is also been chosen as an actuator for generating haptic sensation in a robot-assisted master-salve catheter surgical application scenario.

Another challenge in the area of robot-assisted catheter minimally invasive surgery (MIS) is the interaction force measurement, which is the critical point to artificially generate haptic sensation in the master system. The interaction force between catheter tip and vasculature wall has been studied in depth by Guo et al. [22,23,24]. (Kagawa University, Takamatsu, Japan). However, the force sensor attached to the catheter end effector was inserted into the vasculature of the patient, which not only increases the difficulty of insertion, but also causes infections due to sterilization difficulty. In addition, due to constraints on incision size in MIS, the diameter of the portion of the end effector that enters into the body including all required sensors and/or actuators should be kept less than 10 mm [25]. It is difficult to meet these requirements. Therefore, the sensors located outside of the patient to measure the interaction force method has been presented in this paper to solve this challenge.

In this paper, in order to address these two challenges, we propose a novel MR fluids actuated haptic-based master-slave catheter operating system. The developed MR fluids actuated haptic master system can measure the axial and radial dynamical parameters of the surgeon catheter intervention, which are used as slave site robot control commands. Meanwhile, the haptic sensation of catheter surgery has been generated by controlling the viscosity of MR fluids according to the measured catheter interaction force in the slave site when the surgeon is operating the developed haptic interface. To this end, we developed the robotic catheter navigation system (RNCS). The designed and manufactured RCNS can replicate the dynamic characteristics of a surgeon in the master site. Besides it, the interaction force at the proximal end of the patient-side catheter can also be measured by a force measurement mechanism. Haptic-generating control strategy is dependent on the feedback force that comes from the slave site. The performance and effectiveness of the developed master-slave system has been evaluated in this study.

The remainder of this paper is organized as follows: The developed MR fluids actuated catheter master-slave haptics system is described in Section 2. The master-slave performance evaluation is presented in Section 3. In Section 4, the advantage of haptics experimental evaluation in the in vitro experimental environment has been tested. Finally, discussion and conclusion are presented in Section 5 and Section 6, respectively.

## 2. Materials and Methods

**Figure 1:**
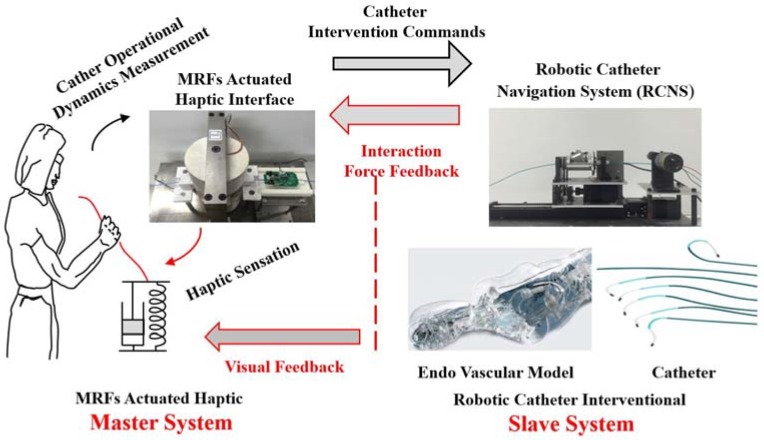
Schematic diagram of the master-slave catheterization system.

This section studies the proposed MRF-actuated haptic-based master-slave system, shown in Figure 1. In this paper, catheter interventional surgery is a kind of minimally invasive surgical procedure which demands high accuracy of insertion velocity control and insertion force control. As far as high accuracy is concerned, the robotic system with autonomous control algorithms has been exploited as the main tool to achieve high accuracy and reliability [4,25]. It is not wise, however, to introduce full autonomous robotic control, for safety and surgical acceptance of the surgical community and patients. Thus, it is acceptable to adopt a semi-autonomous control strategy master-slave robotic-assisted catheter operation system with the characteristics of human-in-the-loop. As shown in Figure 1, the human operator (surgeon) operated the MR fluids actuated haptic master interface with the haptic sensation of catheter interaction situation in the local site, meanwhile the catheter operational dynamics are also measured by the developed dynamic measurement parts integrated into the master haptic interface and is then transmitted to the slave robotic catheter navigation system as the control commands. The robotic catheter intervention slave system repeats the surgeon motion and measures the interaction force between the catheter tip and the vasculature wall by the developed catheter force measurement parts. In addition, the measured force is fed back to the master site as a control input signal to generate a surgeon perceivable haptic sensation by the haptic generating control strategy. Moreover, to improve the performance of the whole master-slave system, in essence not only the haptic sensation generation, but also the visual 3D image visual feedback and force data graph feedback to the surgeon in an actual haptic-based surgical procedure. Although automation technology has been developed to a high level, it is still a human operator (surgeon skills)-centered, robot-assisted, semi-autonomous technology in the medical operational area.

### 2.1. MR Fluids Actuated Haptic Master System

#### 2.1.1. Characteristics of MR Fluids

**Figure 2:**
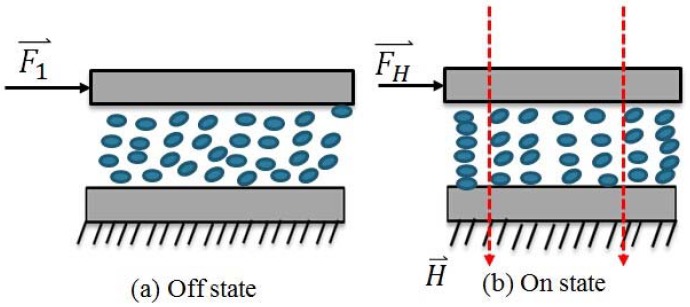
Magnetorheological fluids (MR fluids) effect with external magnetic field or without. (**a**) Off state; (**b**) On state.

Magnetorheological fluids (MR fluids), a kind of smart material, are nonhomogeneous suspension of microsized ferromagnetic particles dispersed in a carrier medium such as silicon oil or water, which undergo a rheological behavior change when an external magnetic field is applied. The mutual interaction among the magnetizable particles form into columns (chains structure) aligned to the direction of the applied external magnetic field [4,26], shown in Figure 2. In essence, the cause of this phenomenon is the viscosity change. The behavior of MR fluid under an external magnetic field can be described as Bingham plastic model with the Equation:

(1) $$\tau =\tau \left(\dot{\gamma },H\right)+|\tau_{y}\left(H\right)|+|\eta \dot{\gamma }|$$

where $\tau \left(\dot{\gamma },H\right)$ is the shear stress which depends on the shear rate $\dot{\gamma }$ and magnetic induction H, $\tau_{y}\left(H\right)$ is the yield stress which depends on the magnetic induction H, $\dot{\gamma }$ is the shear strain rate and η is the viscosity coefficient of MR fluids.

**Figure 3:**
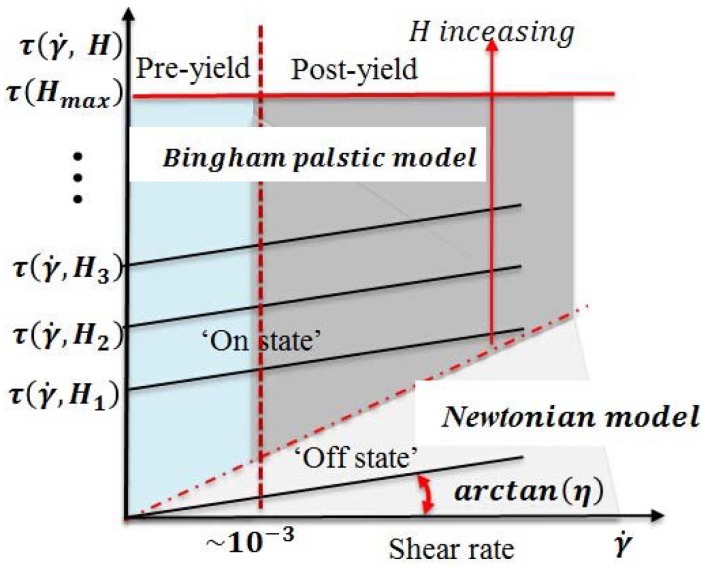
MR fluid’s behaviors upon external electromagnetic field.

During the Bingham plastic model formation process, it can be divided into pre-yield regime and post-yield regime. The Bingham plastic model is a linear behaviour description of the formation process. The shear stress increased with the increased magnetic induction, when $\tau_{y}\left(\dot{\gamma },H\right)<\tau_{y}\left(H\right)$ and $\dot{\gamma }=0$, the behavior of MR fluids in pre-yield regime [26], the chain structures present viscoelastic-like behaviour with some stiffness. This regime is defined as a small elastic deformation region and the strain of the critical point is about 10^−3^ from pre-yield regime to post-yield regime, which is called a yield point [27], shown in Figure 3. When the fluid chain structures deform beyond the yield point $\tau_{y}\left(\dot{\gamma },H\right)>\tau_{y}\left(H\right)$ and $\dot{\gamma }\neq 0$, the fluid is in post yield regime; this behaviour can be described by Equation (1).

**Figure 4:**
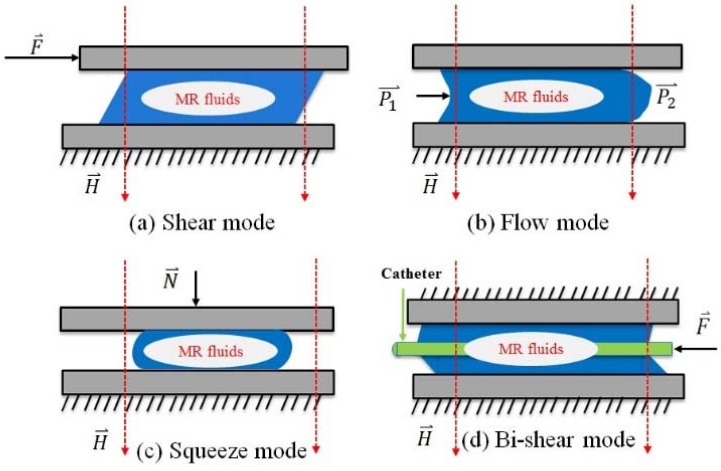
MR fluids working mode, in this paper, Bi-shear mode is adopted. (**a**) Share mode; (**b**) Flow mode; (**c**) Squeeze mode; (**d**) Bi-shear mode.

The degree of resistance depends (chain structure) on the yield stress in MR fluids and has a relationship with the strength of the external magnetic field. MR fluid usually works in three modes. Shear mode is shown in Figure 4a. In this mode, the fluid creates a resistive force against the pole velocity induced by an external force $\vec{F}$. Flow mode is shown in Figure 4b. Magnetic field controls the resistance of flow because of the difference of pressure $∆p=\left(p_{1}-p_{2}\right)$, where p is the pressure. Squeeze mode is shown in Figure 4c. MR fluids resist the normal direction displacement of pole due to a perpendicularly external normal force $\vec{N}$. Figure 4d, bi-shear mode: The fluid builds a resistance force against the catheter going through, which is caused by the external force $\vec{F}$. In this paper, the designed catheter haptic interface is working in bi-shear mode.

#### 2.1.2. Concept Design of MR Fluids Actuated Catheter Haptic Interface

**Figure 5:**
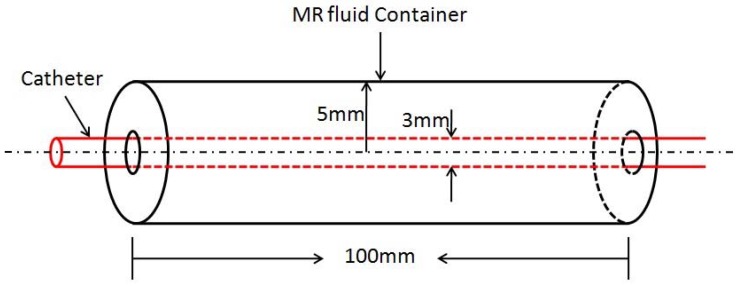
Schematic diagram of the MR fluids container.

In a teleoperated robot-assisted catheter operation scenario, to ensure the safety of the surgery, the haptic sensation obtained in the master site is of critical importance. According to the catheter intervention experienced expert and operational habitation, a kind of force sensation can be used to guide the surgeon with the right speed catheter intervention as well as catheter intervention direction adjustment immediately. Therefore, the key point of the haptic interface design is the design of a high-fidelity force-reflecting system, which makes the surgeon feel like he/she operates the catheter nearby the patient. As has been described above, MR fluids can form a chain structure when applying the external magnetic field to the MR fluids. When inserting the catheter through the chain structure to destroy or change the formed chain structure, a kind of resistive force sensation can be obtained. Moreover, in essence, MR fluid’s chain structure is formed by controlling the viscosity of MR fluids. According to this idea, MR fluids container has been developed as shown in Figure 5.

The container was filled with MR fluids, (MRF-122EG, Lord Crop., Cary, NC, USA), with the characteristic of low permeability, which can never be magnetized during the external magnetic field. The MR fluids container is a cylinder which is in the center of two magnetic poles, its diameter is 10 mm and its length is 100 mm. The catheter goes through the MR fluids, and it is coaxial with the MR fluids container.

**Figure 6:**
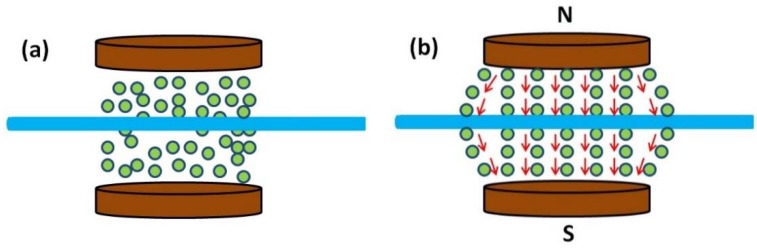

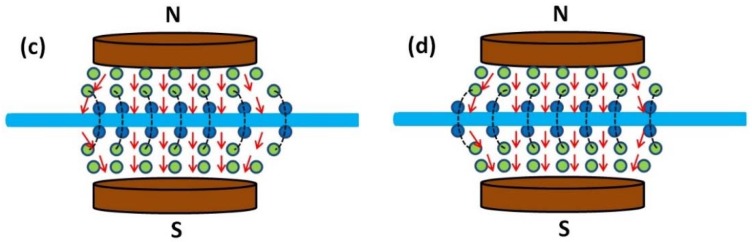
The arrangement state of particles when the magnetic field is applied (**b**–**d**) or not (**a**); (**b**) the catheter was not moved; (**c**) the catheter was moved forward; (**d**) the catheter was moved backward.

The working principles of the catheter intervention through the MR fluids to generate a haptic sensation (show in Figure 6) are discussed as follows:

In the absence of a magnetic field, the particles are statistical law distributed, which can be viewed as a kind of Newtonian fluid, as shown in Figure 6a. When the external magnetic field is applied, the particles changed into the formation of chain-like structures that roughly aligned parallel along the direction of the magnetic field shown in Figure 6b. Figure 6c,d showed that some particles moved along with the catheter, instead of aligning with the external field when the catheter is inserted or retreats through the MR fluids chain structure. During the insertion or retreating of the catheter, the passivity resistance force sensation (kinesthetic haptic sensation) can be perceived by the operator on condition that the precise controlling of the external magnetic field, which matches with the traditional catheter interventional practice that the surgeon actively manipulate a catheter, and then the varied passivity force sensation will be continuously acted on the operator’s fingertips.

**Figure 7:**
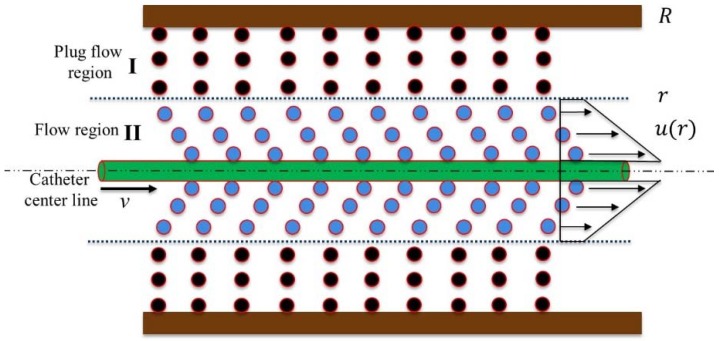
Stress diagram and velocity profile of the Bingham plastic shear flow in a cylinder container.

As mentioned above, MR fluids actuated catheter haptic interface can be realized by controlling the external magnetic field. Therefore, MR fluids viscosity control is the critical important point to generate catheter intervention haptic sensation. In order to obtain precise haptic sensation, the resistance force of MR fluids chain structure change is needed analysis, shown in Figure 7. When the external magnetic field is applied, MR fluids can be viewed as Bingham plastic model. Figure 7 shows the shear stress diagram and velocity profile of the Bingham plastic shear flow in a cylinder container. In region I, the shear stress is lower than the yield stress, there is no shear flow. In region II, the shear stress has exceeded the yield stress; therefore, the fluid flows. So in region I, the shear stress τ is given by:

(2) $$\tau =\tau_{yd}\left(B\right)+\eta \frac{du\left(r\right)}{dr}$$

where *τ_yd_*(*B*) is the magnetic field-dependent yield stress, *B* is magnetic field, *η* is the plastic viscosity, and *du*(*r*)/*dr* is the velocity gradient in the direction of the field. The total resistance force can be written as:

(3) $$F_{R}=F_{yd}+F_{\eta }+F_{f}$$

(4) $$F_{R}=\pi \cdot d\cdot L\cdot \left(\tau_{yb}\left(B\right)+\eta \frac{du\left(r\right)}{dr}\right)+F_{f}$$

where *F_yd_* is the controllable force due to controllable yield stress, *F_η_* is the viscous force, *F_f_* is the mechanical friction force (mainly from seals), *d* is the diameter of the input catheter, *L* is the length of the input catheter which is overwhelmed by the MR fluids.

The viscosity *η* of the carrier fluid is 0.042 Pa·s (MRF-122EG, Lord Crop., Cary, NC, USA). As mentioned in [27], the viscous force decreased two orders of magnitude faster than the controllable force with a large gap size. For reducing the impact of the catheter insertion speed on the generated resistance, in our design the gap between the input catheter and the container inner wall is more than 3 mm in any direction. The second term in Equation (3) can be ignored due to its negligible effect on the estimated values of the resistance force.

In the conventional bedside technique, only the sudden change of the resistance force of patient catheter manipulation can be perceived by the surgeon. So, the control strategy of the catheter haptic sensation generation should consider Weber’s law [4,28]. In Weber’s Law, difference threshold value (DL), the initial intensity of the stimulus $\emptyset_{0}$, ∆∅ is the smallest discriminable intensity of stimulus change, and the constant c is called the Weber Fraction, they are governed by the following Equation:

(5) $$c=\frac{∆\emptyset }{\emptyset_{0}}=\frac{DL}{\emptyset_{0}}$$

What is more, the surgeon can hardly distinguish the collision force from other resistance forces during the catheterization procedure [26]. Tan and Durlach indicated that a just noticeable difference (JND) lay between 100 mN and 200 mN for pinching motions from finger and thumb by constant resistance forces [29]. Considering that the range of resistance force generated by the designed master haptic interface is from 0.014 N to more than 0.5 N (when the applied magnetics flux density is 150 mT). The relationship between the magnetic flux density and resistance force based on different insertion speeds was described in [26]. Therefore, the specific values of resistance force (0.3 N and 0.5 N) were selected as the different levels of haptic feedback to the operator, and the differences between adjacent forces can be noticed by operators.

During the haptic interface design processing, human capability of haptic sensation should be considered, which is determined by two factors: absolute threshold (Absolute Limen) and difference threshold (Difference Limen) according to the psychophysics knowledge. Absolute threshold is defined as the smallest amount of stimulus energy necessary to produce a stimuli sensation, which is dependent on the resolution of the sensory system (shown in Table 1). The difference threshold is defined as the smallest amount of stimulus change required to produce a just noticeable difference (JND) for a discrimination task sensation.

**Figure 8:**
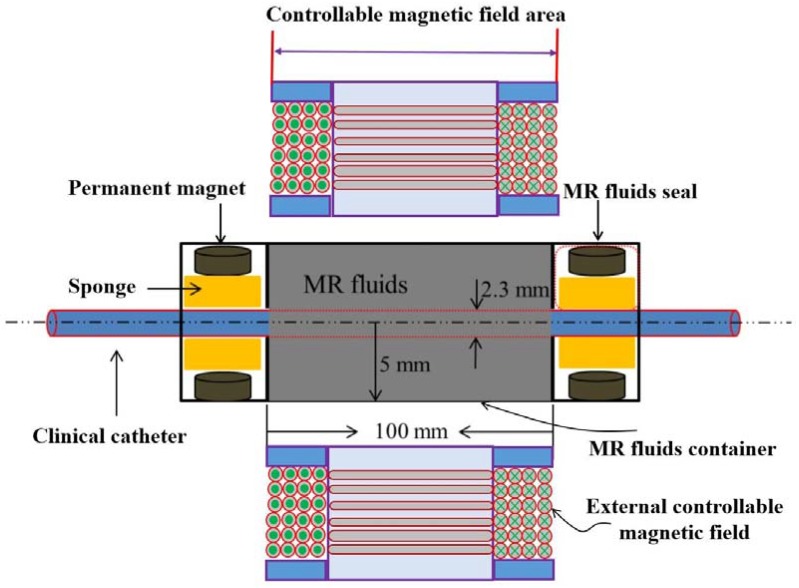
Schematic diagram of MR fluids seal.

According to the requirements of human haptic sensation, the amount of intrinsic system friction force generated by an MR fluids seal should be taken into consideration. Meanwhile, on condition that the increased intrinsic friction force less than the absolute thresholds and difference threshold, there is no effect on human haptic sensation. To meet these requirements and solve MR fluids leakage from hole of the clinical catheter goes through, a kind of MR fluid seal method has been presented and shown in Figure 8. In the design, four small permanent magnets were fixed at both ends of the MR fluids container. Four sponges, which are used to absorb the MR fluids flowing out from the holes of the MR fluids container when MR fluids are not active by an external controllable magnetic field, are placed between the catheter and the permanent magnets. Based on this method, when the external magnetic field is not active and MR fluids are in ‘Newtonian fluid’ situation, the permanent magnets will protect the MR fluids from flowing out from the holes and keep the MR fluids quality constant. In this paper, human haptic sensation is not affected by the MR fluids seal due to the viscosity of the MR fluid controlled by the designed external magnetic field.

#### 2.1.3. External Magnetic Field Design for Controlling of Haptic Sensation

**Figure 9:**
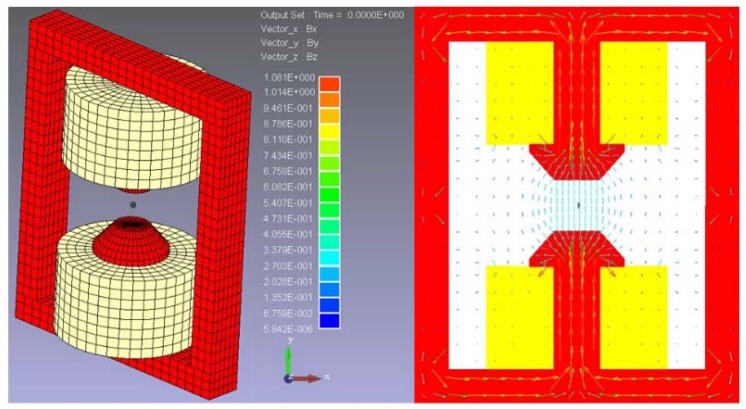
The designed external magnetic field simulation results.

At the external magnetic field design stage, various design parameters including the MR fluids container design, MR fluids seal, coil turns and the supply current, should be considered. Generally, the magnetic flux density shows a non-linear relationship with the applied magnetic field due to hysteresis effect and it cannot increase infinitely with the external magnetic field due to the saturation of coil material [30]. The fluid used as the actuator for generating haptic sensation was MRF-122EG, which can be purchased from the LORD Corporation. According to the LORD technical datasheet [31], this kind of MR fluid exhibits a yield strength of 0−35 kPa for a magnetic field strength of 0−400 kA/m, and with a relative permeability *µ_r_* = 3 − 7. According to the MRF chosen, the above-mentioned MRF haptic working principles and the human operator just notice difference (JND) as well as Weber’s Law, it is a complex system design to obtain the ideal catheter haptic intervention sensation. Therefore, finite element analysis was performed by using the magnetic software; the simulation results are shown in Figure 9 and the dimensions of the designed external magnetic field are shown in Table 2.

#### 2.1.4. The Developed MR Fluids Actuated Catheter Haptic Master System

**Figure 10:**
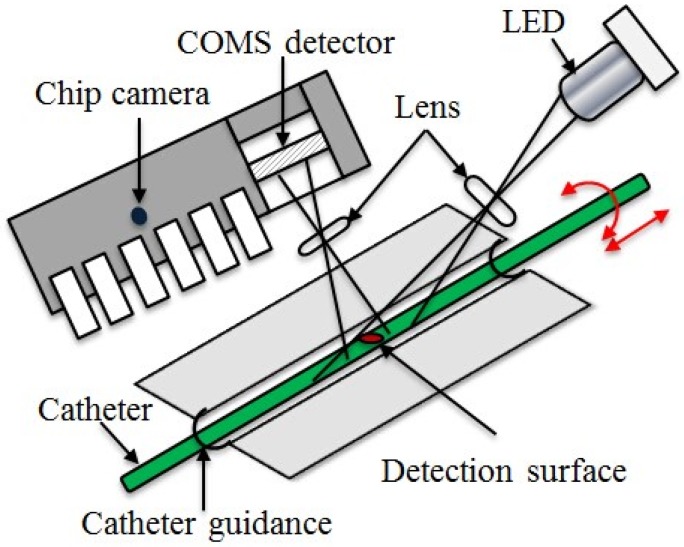
Non-contact catheter interventional kinematics measurement principle.

**Figure 11:**
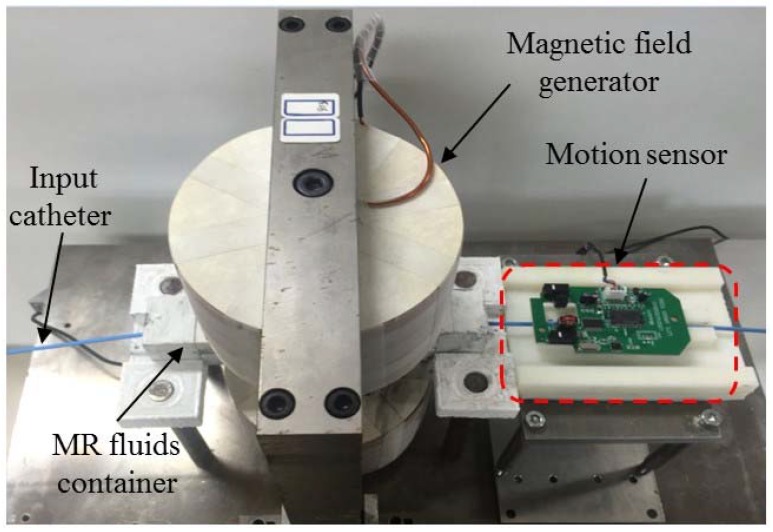
MR fluids actuated catheter haptic master system.

In the master site, the human operator (surgeon) operated the MR fluids actuated haptic master interface with the haptic sensation of the catheter interaction situation in the remote site; meanwhile, the catheter operational dynamics are also measured by the developed dynamics measurement parts integrated in the master haptic interface and are transmitted to the slave robotic catheter navigation system as control commands. Therefore, the catheter haptic master system consists of two parts: One is the human operated catheter intervention dynamics measurement part and the second part is the MR fluids actuated haptic generation. In order to improve the performance of haptic sensation during catheter insertion through the MR fluids actuated haptic generation part. A new kind of non-contact kinematic measurement method for surgeon catheter interventional surgery is presented and shown in Figure 10. The catheter intervention kinematics measured principle is the same with the x direction and y direction displacements measured by the computer mouse. The MR fluids actuated catheter haptic master system is shown in Figure 11.

### 2.2. Robotic Catheter Navigation Slave System

**Figure 12:**
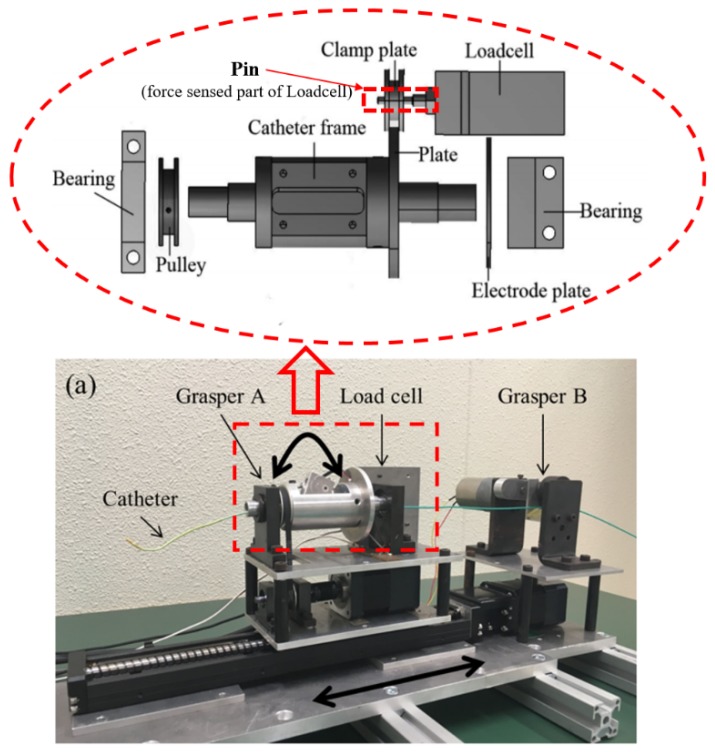
Catheter manipulator and force measurement mechanism.

The Robotic catheter navigation slave system (RCNS) has two functions. One is to manipulate the catheter just like the surgeon catheter intervention, and another is to measure the interaction force during the catheter intervention surgery procedure. Therefore, in the slave system, the catheter manipulator is designed to actuate the patient catheter in transitional ways: To push, pull, and rotate, as shown in Figure 12. The grasper A is controlled by a direct current (DC) gear motor which is fixed on the platform of the linear stage to hold the catheter for axial and radial motions. When the platform moves to the front end, the grasper B (controlled by DC gear motor) will be clamped to keep the position of the catheter and grasper A will be released from the catheter. Then the platform moves back for the next catheter insertion motion. The kinematics of the catheter insertion and retraction process are shown in Figure 12. To prevent the slipping of the catheter, the generated axial gripping force of two graspers is 4 N, and the radial gripping torque of grasper A is 6 Nm, which meets the maximum value of force and torque applied by a surgeon during conventional interventional surgery [32]. Two stepping motors (ASM46AA, Oriental Motor Crop., Tokyo, Japan) with built-in rotor-position sensors are the source of actuation in radial and axial directions, respectively. The high-performance, micro-stepping driver (EN50178, Oriental Motor Crop., Tokyo, Japan) system provides no missteps, even when the load changes suddenly. Load cell (TU-UJ, TEAC Crop., Tokyo, Japan) is applied in this system to measure the axial force information at the distal end of the catheter. The mechanical structure of the force measurement is shown in Figure 8. The accuracy of the axial motion is 0.23 mm (for a 1 m catheter) and the rotation motion is 1.2 deg (for 360 deg) [33].

**Figure 13:**
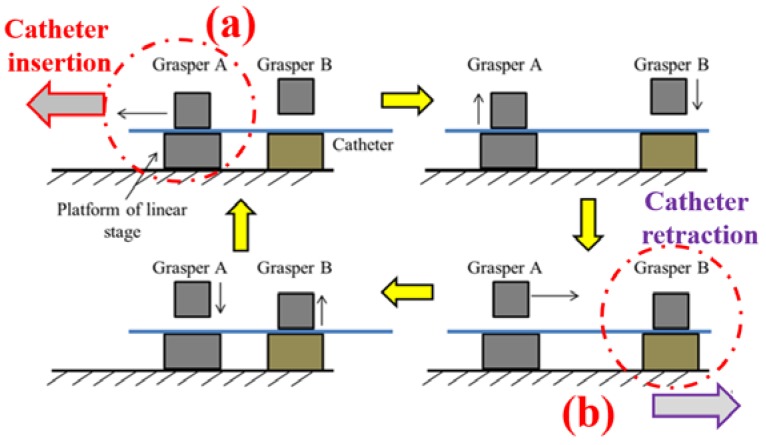
Schematic diagram of catheter insertion. (**a**) Catheter insertion, (**b**) Catheter retraction.

Robot-assisted catheter navigation mechanism and control system are developed instead of the traditional catheter operation method. It needs to be pointed out that the traditional method needs the surgeon doing the clinical catheter surgery to be nearby the patient and to expose themselves to the X-ray. Therefore, the proposed method can solve this issue. Another problem is measuring the interaction force between the catheter and vasculature during catheter intervention. So, the force measurement mechanism has been developed, as shown in Figure 12. The force can be measured by the load cell and is dependent on three conditions: Firstly, the catheter is clamped by the grasper A; secondly, the catheter’s kinematics are the motions of moving forward or backward in the axial direction; thirdly, the grasper B is loosened, as shown in Figure 13a. In this situation, the catheter, catheter frame and catheter plate are fixed together; as shown in Figure 12, the catheter insertion force can be measured, vice versa, the catheter retraction force can be measured just like Figure 13b shows.

### 2.3. Computer Console

The control of the master haptic interface and slave catheter manipulator is achieved through a computer console (3.07 GHz, 4 processors, and 16 GB RAM, TOSHIBA, Tokyo, Japan) via RS-232 serial communication. Control software was implemented using C++ in a windows system, and the data update rate between the master and slave system is 1000 Hz. The motion data of the master haptic interface is transmitted to the computer console by the analogue-to-digital (AD) board (AD 16-16U (PCI), CONTEC, Osaka, Japan), and the motion command is sent to the motor actuators of the slave catheter manipulator via the motion control board (SMC-4DF-PCI, CONTEC Crop., Osaka, Japan). The interaction force of the catheter intervention is measured by a force measurement mechanism attached on the robot catheter navigation system, and it is transmitted to the master site where the different levels of haptic force sensation will be generated.

## 3. System Performance Evaluation

In this section, an evaluation of the lag times of radial and axial motion transmission between the master haptic interface and catheter manipulator is carried out. To quantify the response time of the master-slave system, the input catheter was actuated by the stepping motor with different step motion profiles in axial (speeds are 5 mm/s, 10 mm/s, and 20 mm/s) and radial (15 deg/s, 30 deg/s, and 45 deg/s) directions (up to 200 mm in the axial and 360 deg in the radial directions); the motions were measured by the sensor part of the master haptic interface, and then transmitted to the slave system to actuate catheter manipulator to recopy the catheter intervention happened in the master system. Each speed of insertion or rotation catheter operation was measured 10 times. The rotation motion was repeated in both clockwise and counterclockwise directions.

**Figure 14:**
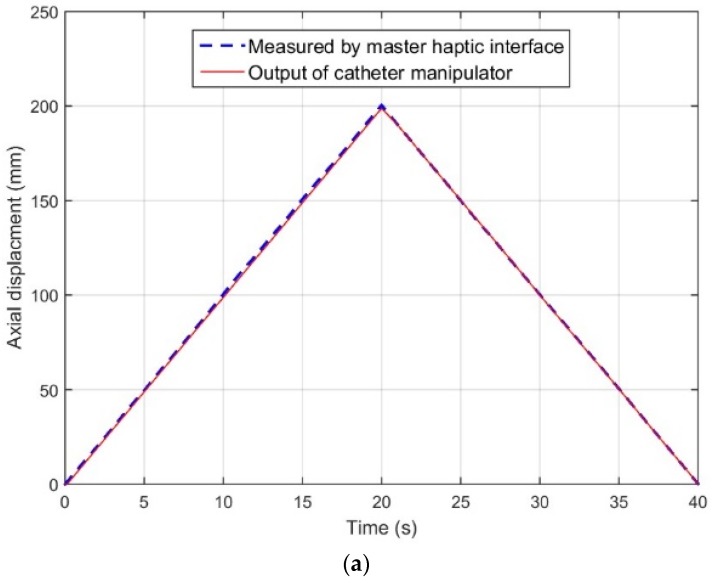

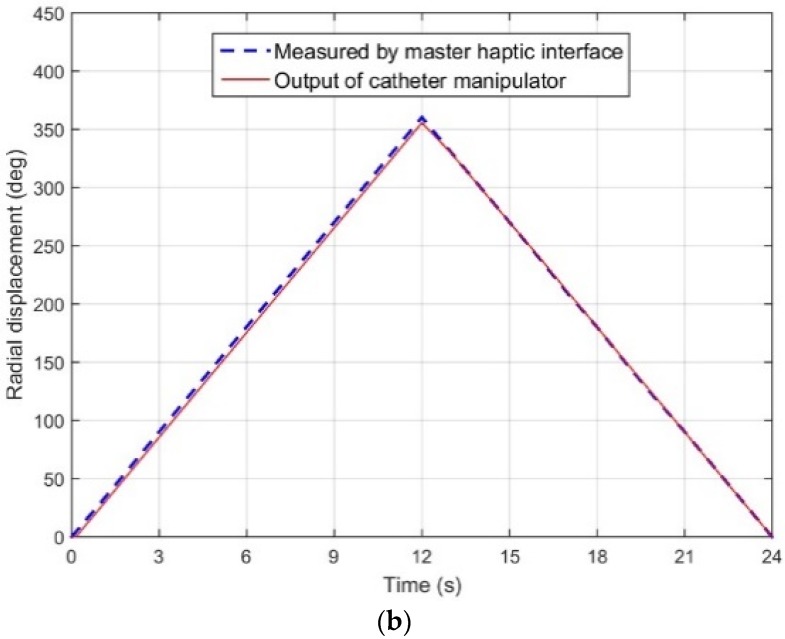
The delay characteristics catheter intervention and rotation between the haptic-based master system and the slave catheter robotic-assisted system. (**a**) Is in axial direction (speed is 10 mm/s) and (**b**) is in radial direction (speed is 30 deg/s).

Figure 14 shows one of the measured delay characteristics of catheter intervention and rotation (the insertion speed is 10 mm/s and the rotation speed is 30 deg/s) separately. Experimental results illustrate that the slave catheter robotic-assisted system can fully recopy the catheter intervention and rotation kinematics. According to the experimental design above, different catheter kinematic experiments have been done and the experimental results are shown in Table 3. In Table 3, the maximum response time is 147 ms with a speed of 45 deg/s catheter rotation, which expresses when catheter with high speed rotation, the slave system cannot follow the master system well immediately. Experimental results also show that the lag time in the radial direction tended to be higher than the axial direction. According to [34], there is an acceptable delay of below 700 ms in the teleoperated robot-assisted surgery scenario; therefore, the time delay between the master interface and the slave catheter navigation system has no effect on the human haptic perception.

## 4. Advantage of Haptics Evaluation In Vitro Experimental Environment

The kinematic characteristics tracking performance of catheter intervention and rotation between the master system and the robot catheter navigation system has been evaluated. In this section, haptic advantage experimental evaluation is illustrated by adopting a robot-assisted teleoperated catheter operation scenario.

### 4.1. Experimental Design

Ten right-handed volunteers, aged from 22 to 28, were recruited by the Guo Laboratory, Kagawa University, to operate the developed MR fluids actuated catheter haptic interface. They had no catheter interventional experience or experience of manipulating the haptics-based master-slave robotic-assisted system. Inexperienced operators were chosen because of their higher kinematic requirements of clinical catheter operation. The volunteers were provided with 20 min of training on the designed system before the experiment. The operators were instructed to operate a 7-Fr clinical used catheter intervention through the master haptic interface to control the slave robotic-assisted catheter operation system with the 5-Fr catheter tip to the disease target. The 5-Fr catheter went through a rigid model of cerebral vascular and then directed the catheter tip into the wide neck aneurysm.

**Figure 15:**
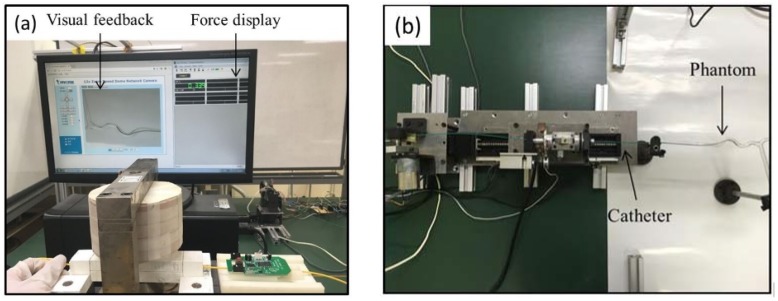
Catheter operation process, (**a**) master system, (**b**) slave system.

In order to simulate 2D fluoroscopy guidance during the experimental process, a web camera was mounted above the vascular phantom to provide the volunteer with vision sensation by image feedback. The processed image was projected on a screen in the master system to help volunteer catheter operation. The catheter operation process is shown in Figure 15. It is noted that inexperienced catheter operation volunteers on the master side will care about hand-eye coordination, which is a key requirement for volunteer’s selection.

**Figure 16:**
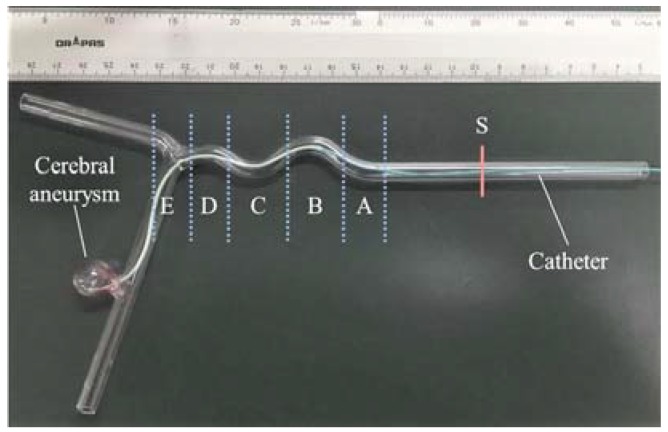
Rigid model of cerebral vascular.

On the slave side, the catheter was inserted into the blood vessel phantom oto the disease target, as shown in Figure 16. According to the physical geometry characteristics of the blood vessel phantom, skill and collision discrimination are needed for the operators when the catheter encountered the tortuous blood vessel. Depending on the difficulty of the catheter manipulation (complexity of vascular phantom, the inner diameter is 5 mm), the collision (between catheter tip and inner wall) and the contact (between the catheter body and inner wall) may happen at the bend position (Section A, Section B, Section C, Section D, Section E). The catheter interventional procedure path is from start position “S” to disease target (a cerebral aneurysm), and the total distance along the axial direction of the phantom is about 170 mm.

**Figure 17:**
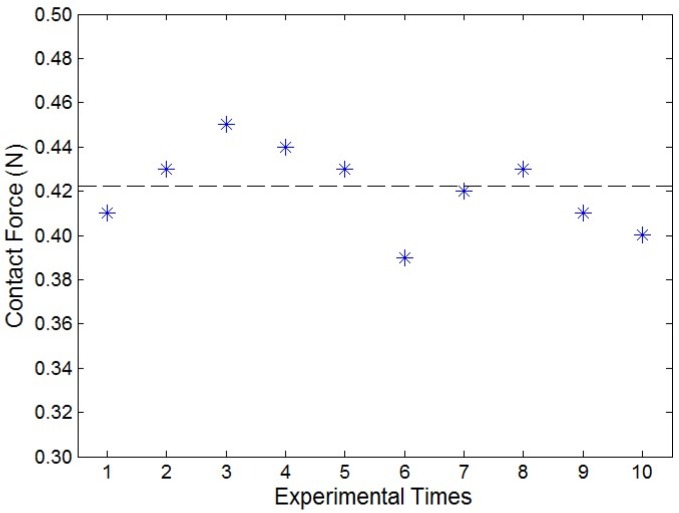
The values of the contact force during 10 times successful catheter operation.

In order to provide haptic sensation to the catheter operation volunteer on the master side, the contact force sensation needs to be measured in the slave system and fed back to the master haptic interface. Here, one kind of catheter operational experimental set was chosen; the catheter insertion speed was planned at 10 mm/s, the rotation kinematics are not considered. The contact force measurement experiments were performed 10 times with successful catheter operation on the disease target point in the slave system by the volunteers; the experimental results are shown in Figure 17. The experiment shows the maximum value of those 10 times was 0.45 N, and the average value was 0.421 N.

According to the above experimental results, the haptic sensation control design in the master side is as follows: Considering the real clinical surgery and the complicity of the force of sensation. Here, in the real time haptic generation control algorithm, when the measured contact force was less than 0.3 N, no haptic sensation was provided to the volunteer; in this situation, the MR fluids actuated catheter haptic interface was in ‘off-situation’, which means MR fluids still in ‘Newtonian fluid’; when the measured contact force is between the ranges of 0.3 N to 0.4 N and less than 0.4 N, it is needed to provide haptic sensation to the volunteer with the corresponding resistance force sensation about 0.3 N or 0.5 N at the external magnetic field intensity was set at 80 mT or 150 mT, according to the experiments done by our group and the published paper [26].

### 4.2. Performance Metrics

Two modes were presented during haptic’s advantage evaluation in this paper. In mode 1, there was no haptic feedback on the master side, just the screen visual feedback. Mode 2 was employed to provide the haptic sensation to volunteers. All volunteers were instructed to complete the same task under mode 1 and mode 2. Each volunteer operated the catheter 10 times in each mode. The performance metrics are defined as follows:

(1) Task completion time: Representing the time required to complete the catheter interventional task.
(2) Contact force: Representing the encountered force of the patient catheter insertion.

### 4.3. Experimental Results

Analysis of variance (ANOVA) was used to analyze the differences between mean values of performance measures and their corresponding procedure; levels of *p* < 0.05 are considered significant.

**Figure 18:**
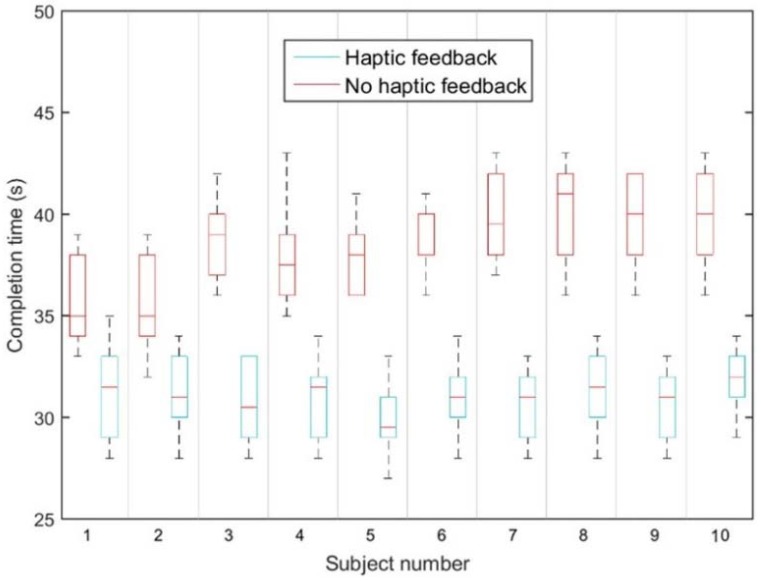
Mean values of task-completion time for each.

Figure 18 illustrates the mean value of task completion times for two modes, while the operators performed each mode 10 times. Each bar shows the average of times taken for each operator to complete both mode 1 and 2. Mean completion times of 10 operators for mode 1 are higher than mode 2. The 2-way ANOVA was conducted to test whether the different modes have a significant effect on the task completion time. For the two modes, we detected a significant difference (*p* = 1.25 × 10^−6^) in the mean completion times, which indicates that the value of the completion time was affected by the provided haptic feedback.

**Figure 19:**
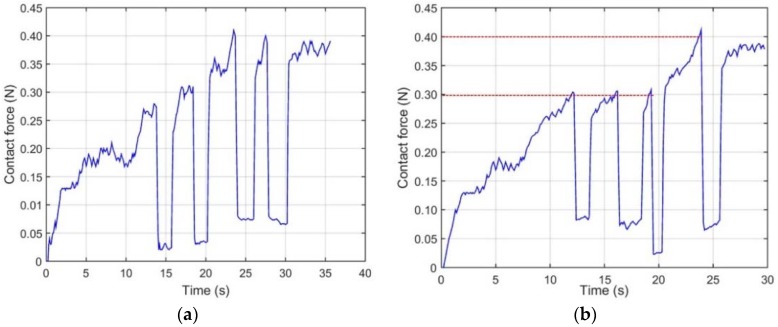
The resistance force trajectories in the slave side: (**a**) The resistance force trajectories under mode 1; (**b**) the resistance force trajectories under mode 2.

Figure 18 shows the experimental example of the contact force of the patient catheter navigation on the axial direction in the slave site. These experimental data recording in Figure 19 are the result of volunteer who experienced half an hour haptic operation or visual catheter operating training in mode 1 and mode 2, respectively.

Generally, when the catheter tip meets the inner wall of the blood vessel phantom, the resistance force will increase. Then, with the length of the path in the phantom increased, the contact force between the catheter body and inner wall will also increase.

In mode 1, the volunteer only used the visual feedback to avoid the collision. In mode 2, the haptic sensation was generated during the whole procedure; the catheter operation volunteer can feel variable haptic sensations based on the feedback changes of the resistance force measured in the slave site by operating the developed MR fluids actuated catheter master haptic interface. It will assist him/her to make decisions in finding suitable directions to reduce the contact force with the help of haptic sensation.

Experimental results show (Figure 19b) that when the contact force existed 0.4 N in the slave site, the haptic sensation was generated. Meanwhile, the catheter operation volunteer on the master site could feel the haptic sensation, so the task finish time was reduced 5 s. The advantage of the haptic sensation in the system has also been verified.

## 5. Discussion

In this paper, MR fluids actuated haptic-based master-slave system has been developed to address the large amount, and the longtime of X-ray radiation during the real surgical procedure by introducing the master-slave robotic-assisted formation. The master-slave system performance has been evaluated and the advantage of haptics in the master-slave catheter operating system has been verified.

Findings from this study indicate that haptic guidance is effective in training based on task completion time in the catheter experiment. Meanwhile, experimental results illustrate that haptic sensation has a larger benefit than visual feedback on the condition that the volunteer of the catheter operation experienced haptic sensation training. As in [32], haptic training is especially helpful when learning motor tasks with complex kinematics, where a haptic sensation removes the need for complex sensorimotor transformations. Moreover, motor skills are difficult to explain and describe visually, which is the reason why visual feedback has a longtime task accomplishment needed than haptic sensation. It also illustrates why studies have shown that learning is better with physical practice as opposed to observational learning. Therefore, it is a benefit for complex, teleoperated, robotic-assisted surgery tasks to provide haptic sensation in the master site.

Despite the fact that extremely promising results have been obtained for the advantages of haptic evaluation, it is important to point out that the study is limited by the fact that in vitro experiments were used to conduct the performance evaluation of the collision protection; the limitations manifest in two ways. Firstly, the used vascular phantom has great rigidity and cannot produce vascular-like deformation during the catheter interventional experiments. Moreover, the curvature of the vascular vessel does not bend the same as the real vessel system, which will lead to an increase of the contact area between the wall of the catheter and the inner wall of the vascular phantom. Secondly, the viscous resistance of the catheter cannot be measured in the slave site, which can be solved by the addition of blood-like lubrication to make the resistance force measure more closely to the real catheter intervention. Therefore, the future research of haptic sensation generation control algorithm will depend on more precise catheter intervention force measurement. Once the more precise force measurement is obtained, more real haptic sensation will be achieved, which can be used in haptic training to improve the skills of the catheter operator.

## 6. Conclusions

In this paper, a MR fluids actuated haptic-based master-slave system has been developed to address the large amount, and the longtime of X-ray radiation during the real surgical procedure by introducing the master-slave robotic-assisted formation. MR fluids-based haptic master catheter haptic interface design procedure has been described in detail to provide haptic sensation to the local site surgeon, and the surgeon’s catheter interventional dynamics have been measured. In the slave site, the slave catheter manipulator mechanism has been designed to do catheter intervention instead of the local site of the surgeon; meanwhile, the interaction force measurement part has been developed to feed back to the surgeon to generate a perceivable haptic sensation for the surgeon.

Some experiments have been of value to the developed master-slave system. The catheter intervention synchronous evaluation experiments between the master and slave system were tested. Also, haptic sensation experiments were evaluated in vitro. The experimental results demonstrated the proposed haptic-based master-slave system can reduce the surgical time and protect the surgeon from X-ray radiation.

The haptic feedback has been utilized to assist the surgeon in decision-making and improving catheter interventional skills. Experimental results also illustrated that haptic feedback was a benefit for providing natural haptic sensation and reducing the human cognitive workload as well as improving the safety of surgery. According to the performance evaluation metrics, we also found that the challenge of improving system transparency in teleoperated robot-assisted catheter intervention surgery can be addressed by adopting haptic feedback control. However, the MR fluid-based haptic interface can only respond to the axial motions of the catheter.

In future, some haptic-based training experiments will be done to further verify the advantages of haptics in complexity motor skill training.

## Figures and Tables

**Table 1:** Sensory resolution and weber fractions for a range of haptic stimuli [4].

Stimulus Dimension	Resolution	Weber Fraction (%)
Force	19 mN	7%
Tangential force	-	16%
Stiffness/compliance	-	15–22%
Friction	-	10–27%
Viscosity	-	19–29%

**Table 2:** The dimensions of the designed external magnetic field.

Parameter Item	Values
Copper Wire Diameter (mm) (AIW)	Ø1.6
Inner Diameter of the Coil (mm)	Ø30
Outer diameter of the coil (mm)	Ø120
Height of Each Coil (mm)	68
Coil Turns (T)	1200
Coil 1 Resistance (Ω)	2.435
Coil 2 Resistance (Ω)	2.448

**Table 3:** Time delay between the master haptic interface and the catheter manipulator.

Speed (Insertion/Rotation)	Time Delay (ms)	
	Mean	Max
5 mm/s	125	129
10 mm/s	121	124
20 mm/s	122	123
15 deg/s	139	144
30 deg/s	141	145
45 deg/s	136	147
